# Targeted Therapy for Metastatic Renal Carcinoma: an Update

**DOI:** 10.15586/jkcvhl.2014.14

**Published:** 2014-10-21

**Authors:** Rodrigo Donalisio da Silva, Diedra Gustafson, Leticia Nogueira, Priya N. Werahera, Wilson R. Molina, Fernando J. Kim

**Affiliations:** 1 Department of Urology, Denver Health Medical Center, Denver, Colorado, USA; 2 Department of Pathology and Department of Bioengineering, University of Colorado Denver, Aurora, Colorado, USA; 3 University of Colorado Cancer Center Denver, University of Colorado Denver, Denver, Colorado, USA

## Abstract

Conventional chemotherapy is associated with poor outcomes in metastatic renal cell carcinoma (RCC). Advances in the understanding of tumor molecular biology and the implementation of new drugs that target these molecular pathways have increased the arsenal against advanced RCC and improved outcomes in these patients. Herein, we briefly describe the latest data on targeted therapies used in the treatment of advanced renal cell carcinoma. Search strategy was performed according to PRISMA guidelines. Abstracts of relevant studies published in PubMed between 2000 and 2014 were analyzed by two authors. Abstracts were selected if they were published in English, data reported was of phase II or III clinical trials, and outcomes followed FDA approval. If consensus between the two authors was achieved, they were included in the review. Key words used were “target therapy” and “metastatic renal cell carcinoma”. The results of the studies analyzed in this review support the benefits of targeted therapy in metastatic RCC. These include improved progression-free survival, overall survival, and quality of life as well as reduced toxicities compared to immunotherapy. The improvement in outcomes in metastatic RCC makes these drugs a preferred option as a primary treatment for these patients.

## Introduction

Renal cell carcinoma (RCC) represents 2-3% of all cancers, with highest incidence occurring in the Western countries (1, 2). In the last two decades, its incidence has been steadily increasing (1). Although a higher incidence of small renal masses are being detected, approximately one third of the patients still have metastatic disease at diagnosis (3, 4). Only a small subset of patients have chosen the historical use of immunotherapy including interleukin-2 (IL-2) and interferon alpha (IFN-α) in the treatment of advanced RCC. These patients have a 5-year survival rate of 6% (5, 6). The moderate efficacy of immunotherapy was also confirmed by a Cochrane meta-analysis using 42 studies (7).

**Figure 1.**
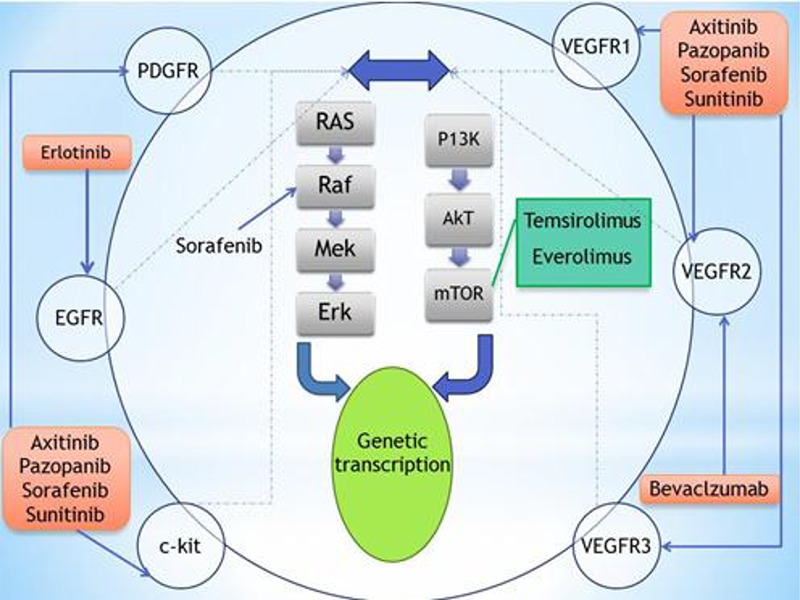
Targeted therapies for metastatic renal cell carcinoma and their mode of action.

Recently, new drugs have emerged in the arsenal of systemic therapy for advanced RCC (Figure 1). A better understanding of the molecular signaling that governs tumor growth and progression has led to the development of molecular therapies targeting the vascular endothelial growth factor (VEGF) and mammalian target of rapamycin (mTOR) pathways, resulting in significant improvement in overall survival and quality of life (3). The objective of this systematic review is to briefly describe the latest data regarding targeted therapies used in the treatment of advanced renal cell carcinoma.

## Methods

### Search Strategy and Study Selection

Search strategy and study selection were performed according to the Preferred Reporting Items for Systematic Reviews and Meta-Analysis (PRISMA) guidelines. Abstracts of relevant studies and clinical trials from PUBMED/MEDLINE (2000 to 2014) were analyzed by two authors and were included if both agreed with the selection. A third author was consulted when the two authors disagreed. After abstract selection, all manuscripts were revised and were only included if it met the selection criteria and if consensus was achieved by the authors.

The key words used were “target therapy” and “metastatic renal cell carcinoma”. The terms identified included names of following therapies: *Sunitinib, Sorafenib, Pazopanib, Axitinib, Cediranib, Everolimus, Temsirolimus*, *Bevacizumab*, and *Erlotinib*.

Study inclusion criteria included contemporary articles published in English after 2000 that reported data of phase II and III Clinical Trials and outcomes followed FDA approval. A total of 40 studies were eligible for review.

### Data Extraction and Analysis

Variables collected from eligible studies were: study name, period of the study, molecular targets of the drug, FDA approval status, indication of treatment, recommended dosage of the drug, and safety and efficacy of the drug. Efficacy was evaluated by the Overall survival (OS), progression free survival (PFS), and time to progression (TTP) as defined by the FDA Center for Drug Evaluation and Research. Safety was evaluated by the severity of adverse events defined by the Common Toxicity Criteria (CTC).

## Evidence synthesis

### VEGF Targeted Therapies

Angiogenesis is critical for tumor growth and progression, especially in solid tumors with vast vascularization such as RCC. Vascular endothelial growth factor and its receptor (VEGF/VEGFR) mediate VEGFR regulation of vessel permeability, endothelial cell activation, survival, proliferation, invasion, and migration. VEGFR and PDGFR pathways exhibit tyrosine kinase activity and activate downstream signaling pathways as the Raf/MEK/ERK (8). During angiogenesis, Raf is key in regulating endothelial cell survival by controlling apoptosis pathways (9). Several drugs have been developed to target this pathway and control tumor angiogenesis. A list of novel therapeutics targeting the angiogenesis/VEGF pathway is summarized in Table 1.

**Table 1:** Angiogenesis/VEGF inhibitors: dose, molecular target and PFS outcome.

Therapy	Dose	Target	Line of Therapy	Study	PFS (months)	Ref
Sorafenib	Oral; 400mg BID	Raf-1 serine/threonine kinase, B-Raf, VEGFR-2, PDGFR. C_KIT	Second Linecyto	Sorafenib v. Placebo	5.5 v. 2.8*	(10)
Sunitinib	Oral; 50mg qd	VEGFR1-3, c-KIT, FLT3 PDGFR	First Line	Sunitinib v. IFN	11 v. 5*	(11)
Pazopanib	Oral; 800mg qd	VEGFR1-3; RET, c-kit	First Line	Pazopanib v. Sunitinib	8.4 v. 9.5	(15)
			First Line	Pazopanib v Placebo	11.1 v. 2.8*	(14)
			Second Line	Pazopanib v Placebo	7.4 v. 4.2*	(17)
Axitinib	Oral; 5mg tid	VEGFR1	First Line	Axitinib v. Sorafenib	10.1 v. 6.5*	(19)
			Second Linecyto, vegf, mtor	Axitinib v. Sorafenib	6.7 v. 4.7*	(18)
Cediranibi	Oral; 45mg tid	VEGF1-3	First Line	Cediranib v. Placebo	12.1 v. 2.8*	(22)
Bevacizumab-IFN	IV; 10mg/Kg 2/2weeks	VEGF	First Line	Bevacizumab-IFN v IFN	8.5 v. 5.2*	(26)
				Bevacizumab-IFN v IFN	10.2 v. 5.4*	(25)
Bevacizumab-Erlotinibi	Oral; 150mg qd	EGFR tyrosine kinase	First Line	Bevacizumab-Erlontinib v Bevacizumab	9.9 v. 8.5	(28)

PFS, progression free survival; i, investigational drug; cyto, post-cytokine; vegf, post-VEGF; mtor, post-mTORi; ^*^ statistically significant.

### Sorafenib

Sorafenib is an oral multi-tyrosine kinase inhibitor with activity against Raf-1 serine/threonine kinase, B-Raf, vascular endothelial grow factor receptor 2 (VGEFR-2), PDGFR, and c-kit. A phase III trial (TARGET trial) showed a significantly longer PFS with sorafenib compared to placebo (5.5 vs. 2.8 months; p < 0.001). Moreover, partial responses were significantly higher (10%) in those patients treated with sorafenib compared to 2% of those treated with placebo (P<0.001). Cross over was performed in patients of the placebo group which presented a reduced risk of death. 16 months after crossover, the overall survival in the sorafenib treated cohort was 17.8 months compared to 15.2 months for the patients initially treated with placebo (p < 0.146). The estimated overall survival for the placebo-treated patients was 14.3 months.

Sorafenib is considered a second line therapy and the suggested dose is 800 mg a day. Adverse effects were skin rash, hand-foot skin reaction, and fatigue. Discontinuation of the treatment was required in 9% of patients, and no deaths were reported due to toxicity of the treatment (10). Sorafenib was the first anti-angiogenic multi-tyrosine kinase inhibitor for mRCC approved by the FDA (2005).

### Sunitinib

Sunitinib is also an inhibitor of VEGFR1-3, c-kit, FLT-3 and PDGFR. Sunitinib has direct antitumor and anti-angiogenic activity (10, 11). This drug was approved by the FDA in 2006 and is now considered a first-line therapy for mRCC. It is orally administered with the recommended daily dose of 50 mg/day by a schedule 4/2.

In a phase III trial comparing sunitinib to interferon, the sunitinib arm showed doubled progression-free survival (PFS), improving PFS from 5 months with interferon to 11 with sunitinib as monotherapy. The objective response rates were 47% and 12% for sunitinib and interferon-α, respectively (P <0.001) and the median overall survival was 26.4 months for sunitinib and 21.8 months for interferon-α (P = 0.051) (12). Moreover, access expanded globally, and another phase III trial was designed to provide sunitinib on relatively unselected or trial-ineligible patients with brain metastases and poor ECOG performance status. Treatment with sunitinib demonstrated a PFS of 10.9 months and median overall survival of 18.4 months with similar overall survival in patients with and without prior cytokine therapy. Sunitinib did not present high severity adverse events, but hypertension, fatigue, diarrhea, and hand-foot syndrome were described during treatment with this drug. Sunitinib was compared with IFN-α regarding quality-adjusted time without symptoms of disease progression or toxicity of treatment (Q-TWiST score). Sunitinib resulted in better clinical efficacy and quality-of-life outcomes compared with IFN-α for mRCC patients (13).

### Pazopanib

Pazopanib is a second generation, orally administered multi-target tyrosine kinase receptor inhibitor that blocks VEGFR1-3, RET, and c-kit receptors (^11-13^). A randomized phase III trial comparing pazopanib with placebo showed a significant improvement in PFS and tumor response in treatment-naïve metastatic RCC patients (54%) and previously cytokine-treated patients (46%). Compared to placebo, the overall PFS was 9.2 months for the pazopanib group vs 4.2 months for placebo patients (HR: 0.46; 95% CI: 0.34-0.62; p<0.0001). In the treatment-naïve subpopulation, PFS was 11.1 months vs 2.8 months (HR: 0.40; 95% CI: 0.27-0.60; p<0.0001) for pazopanib and placebo, respectively. In patients pretreated with cytokine, PFS was 7.4 months vs 4.2 months (HR: 0.54; 95% CI: 0.35-0.84; p<0.001) for pazopanib and placebo, respectively (14).

Another non-inferiority randomized phase III trial compared pazopanib with sunitinib. PFS and OS of pazopanib were not inferior to sunitinib, and quality of life with pazopanib was statistically better than sunitinib in those patients PE (15). Pazopanib demonstrated acceptable safety and tolerability even though it has been associated with liver toxicity. Common adverse events reported with pazopanib were hair color changes, nausea, anorexia, and vomiting while Grade 3-4 toxicity effects were hypertension, diarrhea, and liver toxicity PEVuZE (14). Pazopanib was approved by the FDA in 2009. It is considered as a first-line treatment and an option as a second-line treatment in previously cytokine-treated patients (16, 17). Pazopanib is usually administered orally at 800 mg daily.

**Table:** Table 2: m-TOR inhibitors: dose, molecular target and PFS outcome.

Therapy	Dose	Target	Line of Therapy	Study	PFS (months)	Ref
Temsirolimus	IV; 25mg weekly	mTOR; HIF1-2; VEGF	First Line	Temsirolimus v IFN	10.9 v. 7.3*	(38)
			Second Linevegf	Temsirolimus v Sorafenib	4.3 v. 3.9	(45)
Everolimus	Oral; 10mg Qd	mTOR; HIF1;VEGF	Second/Third Linevegf	Everolimus v. Placebo	4.9 v. 1.9*	(36)

PFS, progression free survival; vegf, post-VEGF; ^*^ statistically significant.

### Axitinib

Axitinib is another second-generation inhibitor of VEGFR-1 which also has minimal effect on other targets. Axitinib is a second-line therapy option in cytokine-refractory metastatic RCC. A Phase III clinical trial (18) that compared axitinib and sorafenib in 723 patients who were previously treated unsuccessfully with cytokine or VEGF inhibitors showed a median PFS of 6.7 months for the axitinib group and 4.7 months for the sorafenib group (p<0.0001). The OS was 29.9 months with a TTP of 15.7 months.

The overall response rate was 22.6%, and the median duration of response was 17.5 months. The adverse events of axitinib included diarrhea, hypertension, fatigue, dysphonia, and hand-foot syndrome (19). Grade 3 to 4 adverse events included hand-foot syndrome, fatigue, hypertension, dyspnea, diarrhea, dehydration, and hypotension. Axitinib was approved by the FDA in 2012. Its potency is 50 to 450 times greater than the first-generation VEGFR inhibitors (20, 21). The recommended dose of axitinib is 5.0 mg twice a day (18).

### Cediranib

Cediranib is an ATP-competitive inhibitor of receptor tyrosine kinases (RTKs) related to VEGF1-3 (11). A phase II trial compared the efficacy of cediranib with placebo in patients with metastatic or recurrent clear cell RCC who had not previously received a VEGF signaling inhibitor. Partial responses were achieved in 34% patients, and 47% experienced a stable disease. PFS significantly improved when compared to placebo with median 12.1 versus 2.8 months (p = 0.017) (22). In addition, more than 50% of patients who achieved a partial response with cediranib experienced responses lasting more than a year. The most common adverse effects in patients were diarrhea, fatigue, hypertension, and dysphonia (23). The recommend dose is 45 mg/day. Cediranib is still an investigational drug under the FDA.

### Bevacizumab

Bevacizumab is a humanized recombinant IgG monoclonal antibody that binds to VEGF-A, increasing vascular permeability and reducing proliferation and migration of endothelial cells. The AVOREN (phase III) double-blind trial randomized 649 naïve patients to receive bevacizumab (10 mg/kg every 2 weeks) plus IFN-α (9 MUI) or placebo and IFN-α. The median overall response (OR) and stable disease in the bevacizumab plus IFN-α versus placebo plus IFN-α arms were 31 and 46% versus 13 and 50%, respectively. PFS was significantly longer in the bevacizumab and IFN-α (10.2 versus 5.4 months; p<0.0001), but only in good-risk and intermediate-risk patients. In poor-risk patients, bevacizumab did not present any benefits (24).

After progression, crossover was performed and the median OS was 23.3 months for bevacizumab-IFN-α vs 21.3 months for IFN-α alone (p=0.336) (25). Fatigue, asthenia, and proteinuria were the most common grade 3 toxicities (11, 26). FDA approved bevacizumab in 2009 at 10 mg/kg IV every 2 weeks in combination with IFN-α.

Another phase III (CALGB 90206)(26) randomized trial enrolled 732 previously untreated metastatic RCC patients for bevacizumab (10 mg/kg each 2 weeks) plus IFN-α (9 million U/3 times weekly) versus IFN-α monotherapy. PFS was 8.5 months for the combination compared to 5.2 months for INF-α alone. After crossover, median OS was 18.3 for the combination compared to 17.4 for IFN-α alone. OR with bevacizumab plus IFN-α was higher compared to IFN monotherapy (25.5 vs 13.1% p< 0.0001). The combination therapy was associated with higher grade 3 to 4 hypertension (HTN), anorexia, fatigue, and proteinuria.

### Erlotinib

Erlotinib inhibits the tyrosine kinase domain of epidermal growth factor receptor (EGFR), leading to the inhibition of EGFR auto-phosphorylation and downstream signaling (27). Erlotinib demonstrated encouraging activity in renal cell carcinoma when associated with bevacizumab in a phase II trial (28). 63 patients with metastatic clear-cell RCC were treated with bevacizumab at 10 mg/kg every 2 weeks and erlotinib at 150 mg/daily. Objective responses were achieved in 25% of the patients, disease was stable in 61% after 8 weeks, and survival at 18 months was 60%. Another randomized double-blind phase II trial compared the combination of erlotinib and bevacizumab with bevacizumab alone. Combined therapy did not provide additional benefits when compared to bevacizumab alone (29).

The combination of erlotinib with sirolimus in metastatic RCC did not show benefits when compared to a single agent in a phase II trial (30). 25 patients previously treated with sunitinib and/or sorafenib were evaluated and included. Partial responses or complete responses were observed; however, stable disease was noted in 21.8% of patients in 46 months. The progression-free survival and overall survival were 12 and 40 weeks respectively. Currently, Erlotinib is not approved by the FDA for the treatment of metastatic RCC.

## Mammalian Target of Rapamycin (mTOR) Inhibitors

Another signaling pathway that is critical for cellular growth, proliferation, and angiogenesis is the mammalian target of rapamycin (mTOR) pathway (31). This pathway is more significantly mutated in clear-cell RCC, high-grade tumors, and tumors with poor prognostic features (32, 33). A list of novel therapeutics targeting the mTOR pathway is summarized in Table 2.

### Everolimus

Everolimus is an mTOR inhibitor used in the treatment of VEGF- refractory disease. A phase II trial was conducted using everolimus at a daily dose of 10 mg for a 28-day cycle in 41 patients with metastatic RCC who were previously treated with one therapy at most. Median progression-free survival of 11.2 months and median overall survival of 22.1 months was reported (34). Partial responses were observed in 5 patients, stable disease lasting 3 months was reported in 27 patients, and stable disease lasting 6 months was reported in 21 patients.

Another phase II trial in metastatic RCC patients who hadn’t received previous treatment or who had failed RCC treatment on sunitinib and/or sorafenib demonstrated anti-tumoral activity with the combination of everolimus and bevacizumab. Bevacizumab was given at 10 mg/kg intravenously every 2 weeks and everolimus at 10 mg per day, orally. The median PFS in previously untreated patients was 9.1 months and 7.1 months in previously treated patients (35).

A placebo-controlled phase III trial was designed with everolimus as a second-line therapy for advanced clear cell carcinoma refractory to sunitinib, sorafenib, or both agents. 410 patients were randomized to receive everolimus or placebo. Patients were stratified according MSKCC (Memorial Sloan-Kettering Cancer Center) prognostics score and whether they had previously received one or two VEGF receptor tyrosine kinase inhibitors. PFS was significantly prolonged for everolimus by 4.9 months when compared to 1.87 months with placebo (36). Common adverse effects reported were stomatitis, rash, diarrhea, and non-infectious pneumonitis. Everolimus was approved by the FDA in 2009 as an option for advanced RCC patients who had failed treatment with VEGF therapy. The usual dose is 10 mg once daily (37).

### Temsirolimus

Temsirolimus is a specific inhibitor of mTOR and inhibits tumor angiogenesis by reducing synthesis of VEGF. Temsirolimus and IFN-α were used in a phase I/II Trial (38) for advanced RCC. 71 RCC patients were eligible and the recommended doses for temsirolimus was 15 mg and IFN-α was 6 million units. Among patients who received the recommended dose, 8% achieved partial response, 36% had a stable disease for 24 weeks, and the median overall progression-free survival was 9.1 months.

A phase III trial with 626 advanced and poor prognosis patients established that temsirolimus in combination with interferon did not improve survival (39). Overall survival medians in the interferon, temsirolimus, and combination groups were 7.3, 10.9, and 8.4 months, respectively. Monotherapy with temsirolimus showed longer overall survival and progression-free survival (P<0.001) than patients who received interferon alone (P<0.001). The median OS of temsirolimus and IFN-α as monotherapies were 10.9 and 7.3 months respectively. The median PFS time for the temsirolimus was 5.5 months compared with 3.1 months on IFN-α (p = 0.001).

Common adverse events were rash, peripheral edema, hyperglycemia, and hyperlipidemia in the temsirolimus group whereas asthenia was more significant in the interferon group. Grade 3 or 4 toxicity occurred in almost 90% of patients in the combination therapy. Temsirolimus was approved by the FDA in 2007 for advanced/metastatic RCC patients with three or more poor prognostic features. The standard dose is 25 mg IV/weekly.

## Non-clear cell histology

Presently, there is a lack of phase III trials on systemic treatment of patients with non-clear cell RCC. Small studies for papillary type 1 and 2 were performed with sunitinib and everolimus, but none of them were prospectively randomized (40, 41). A phase II trial in patients with papillary RCC treated with foretinib (multikinase inhibitor) showed a median PFS of 9.3 months and 13% response rate. The presence of germ line MET mutation was a strong predictor of a response (42). There is a lack of data to support systemic therapy in patients with collecting-duct subtype. These tumors have been included in prospective trials but with smaller numbers of patients, invalidating any type of analysis (43).

## Cytoreductive Nephrectomy in target therapy era

Cytoreductive nephrectomy has been shown to extend overall patient survival in the multimodal treatment of metastatic RCC comparing immunotherapy alone or combined with cytoreductive nephrectomy (44). In this target therapy era, it’s likely to remain part of the treatment and is recommended when possible. Complete removal of metastasis contributes to improved clinical prognosis and should be considered when feasible (2).

## Conclusion

A better understanding of the tumor biology and the development and approval of multiple targeted agents for treatment of advanced RCC enables improved survival in patients with metastatic RCC. The standard of care in metastatic RCC is use of drugs that target VEGF and mTOR pathways. The third generation of tyrosine kinase inhibitors appears to have similar or superior efficacy as well as lower toxicity than existing agents.

Compared to previous systemic therapies, these drugs showed evident clinical benefits. They increase progression-free overall survival and improve the quality of life, but complete responses have been rarely noted. Some questions have yet to be answered and demand more debate. The most efficacious sequence of therapies and time to start a second-line agent (before or not progression of the disease) should be addressed in further studies.

## References

[1] Guidelines on Renal Cell Carcinoma [Internet] (2014). European Urology Association.

[2] Ljungberg B, Cowan NC, Hanbury DC, Hora M, Kuczyk MA, Merseburger AS (2010). EAU guidelines on renal cell carcinoma: the 2010 update.. Eur Urol.

[3] Najjar YG, Rini BI. (2012). Novel agents in renal carcinoma: a reality check.. Ther Adv Med Oncol.

[4] Stadler WM. (2005). Targeted agents for the treatment of advanced renal cell carcinoma.. Cancer.

[5] Berg WJ, Divgi CR, Nanus DM, Motzer RJ. (2000). Novel investigative approaches for advanced renal cell carcinoma.. Semin Oncol.

[6] Dorff TB, Goldkorn A, Quinn DI. (2009). Targeted therapy in renal cancer.. Ther Adv Med Oncol.

[7] Coppin C, Porzsolt F, Awa A, Kumpf J, Coldman A, Wilt T. (2005). Immunotherapy for advanced renal cell cancer.. Cochrane Database Syst Rev.

[8] Rak J, Kerbel RS. (2001). Ras regulation of vascular endothelial growth factor and angiogenesis.. Methods Enzymol.

[9] Hilger RA, Scheulen ME, Strumberg D. (2002). The Ras-Raf-MEK-ERK pathway in the treatment of cancer.. Onkologie.

[10] Ratain MJ, Eisen T, Stadler WM, Flaherty KT, Kaye SB, Rosner GL (2006). Phase II placebo-controlled randomized discontinuation trial of sorafenib in patients with metastatic renal cell carcinoma.. J Clin Oncol.

[11] Vakkalanka BK, Bukowski RM. (2008). Novel drugs for renal cell carcinoma.. Expert Opin Investig Drugs.

[12] Al-Marrawi MY, Rini B. (2011). Pazopanib for the treatment of renal cancer.. Expert Opin Pharmacother.

[13] Vasudev NS, Larkin JM. (2011). Tyrosine kinase inhibitors in the treatment of advanced renal cell carcinoma: focus on pazopanib.. Clin Med Insights Oncol.

[14] Sternberg CN, Davis ID, Mardiak J, Szczylik C, Lee E, Wagstaff J (2010). Pazopanib in locally advanced or metastatic renal cell carcinoma: results of a randomized phase III trial.. J Clin Oncol.

[15] Motzer RJ, Hutson TE, Cella D, Reeves J, Hawkins R, Guo J (2013). Pazopanib versus sunitinib in metastatic renal-cell carcinoma.. N Engl J Med.

[16] Bukowski RM. (2012). Third generation tyrosine kinase inhibitors and their development in advanced renal cell carcinoma.. Front Oncol.

[17] Keisner SV, Shah SR. (2011). Pazopanib: the newest tyrosine kinase inhibitor for the treatment of advanced or metastatic renal cell carcinoma.. Drugs.

[18] Rixe O, Bukowski RM, Michaelson MD, Wilding G, Hudes GR, Bolte O (2007). Axitinib treatment in patients with cytokine-refractory metastatic renal-cell cancer: a phase II study.. Lancet Oncol.

[19] Rini BI, Escudier B, Tomczak P, Kaprin A, Szczylik C, Hutson TE (2011). Comparative effectiveness of axitinib versus sorafenib in advanced renal cell carcinoma (AXIS): a randomised phase 3 trial.. Lancet.

[20] Carmichael C, Lau C, Josephson DY, Pal SK. (2012). Comprehensive overview of axitinib development in solid malignancies: focus on metastatic renal cell carcinoma.. Clin Adv Hematol Oncol.

[21] Sonpavde G, Hutson TE, Rini BI. (2008). Axitinib for renal cell carcinoma.. Expert Opin Investig Drugs.

[22] Sridhar SS, Mackenzie MJ, Hotte SJ, Mukherjee SD, Tannock IF, Murray N (2013). A phase II study of cediranib (AZD 2171) in treatment naive patients with progressive unresectable recurrent or metastatic renal cell carcinoma.. A trial of the PMH phase 2 consortium. Invest New Drugs.

[23] Mulders P, Hawkins R, Nathan P, de Jong I, Osanto S, Porfiri E (2012). Cediranib monotherapy in patients with advanced renal cell carcinoma: results of a randomised phase II study.. Eur J Cancer.

[24] Escudier B, Bellmunt J, Négrier S, Bajetta E, Melichar B, Bracarda S (2010). Phase III trial of bevacizumab plus interferon alfa-2a in patients with metastatic renal cell carcinoma (AVOREN): final analysis of overall survival.. J Clin Oncol.

[25] Trump DL. (2012). Commentary on “comparative effectiveness of axitinib vs. sorafenib in advanced renal cell carcinoma (AXIS): a randomized phase 3 trial.” B.I. Rini, B. Escudier, P. Tomczak, A. Kaprin, C. Szczylik, T.E. Hutson, M.D. Michaelson, V.A. Gorbunova, M.E. Gore, I.G. Rusakov, S. Negrier, Y.C. Ou, D. Castellano, H.Y. Lim, H. Uemura, J. Tarazi, D. Cella, C. Chen, B. Rosbrook, S. Kim, R.J. Motzer: Lancet 2011;378:1931-9 [Epub;2011, November 4].. Urol Oncol.

[26] Rini BI, Halabi S, Rosenberg JE, Stadler WM, Vaena DA, Ou SS (2008). Bevacizumab plus interferon alfa compared with interferon alfa monotherapy in patients with metastatic renal cell carcinoma: CALGB 90206.. J Clin Oncol.

[27] Tang PA, Tsao MS, Moore MJ. (2006). A review of erlotinib and its clinical use.. Expert Opin Pharmacother.

[28] Hainsworth JD, Sosman JA, Spigel DR, Edwards DL, Baughman C, Greco A. (2005). Treatment of metastatic renal cell carcinoma with a combination of bevacizumab and erlotinib.. J Clin Oncol.

[29] Bukowski RM, Kabbinavar FF, Figlin RA, Flaherty K, Srinivas S, Vaishampayan U (2007). Randomized phase II study of erlotinib combined with bevacizumab compared with bevacizumab alone in metastatic renal cell cancer.. J Clin Oncol.

[30] Flaig TW, Costa LJ, Gustafson DL, Breaker K, Schultz MK, Crighton F (2010). Safety and efficacy of the combination of erlotinib and sirolimus for the treatment of metastatic renal cell carcinoma after failure of sunitinib or sorafenib.. Br J Cancer.

[31] Sonpavde G, Choueiri TK. (2012). Biomarkers: the next therapeutic hurdle in metastatic renal cell carcinoma.. Br J Cancer.

[32] Cho DC, Atkins MB. (2011). Future directions in renal cell carcinoma: 2011 and beyond.. Hematol Oncol Clin North Am.

[33] Pinto Marín A, Redondo Sánchez A, Espinosa Arranz E, Zamora Au-ón P, Castelo Fernández B, González Barón M. (2012). mTOR pathway inhibition in renal cell carcinoma.. Urol Oncol.

[34] Amato R. (2011). Everolimus for the treatment of advanced renal cell carcinoma.. Expert Opin Pharmacother.

[35] Hainsworth JD, Spigel DR, Burris HA, Waterhouse D, Clark BL, Whorf R. (2010). Phase II trial of bevacizumab and everolimus in patients with advanced renal cell carcinoma.. J Clin Oncol.

[36] Motzer RJ, Escudier B, Oudard S, Hutson TE, Porta C, Bracarda S (2008). Efficacy of everolimus in advanced renal cell carcinoma: a double-blind, randomised, placebo-controlled phase III trial.. Lancet.

[37] Voss MH, Molina AM, Motzer RJ. (2011). mTOR inhibitors in advanced renal cell carcinoma.. Hematol Oncol Clin North Am.

[38] Motzer RJ, Hudes GR, Curti BD, McDermott DF, Escudier BJ, Negrier S (2007). Phase I/II trial of temsirolimus combined with interferon alfa for advanced renal cell carcinoma.. J Clin Oncol.

[39] Hudes G, Carducci M, Tomczak P, Dutcher J, Figlin R, Kapoor A (2007). Temsirolimus, interferon alfa, or both for advanced renal-cell carcinoma.. N Engl J Med.

[40] Koh Y, Lim HY, Ahn JH, Lee JL, Rha SY, Kim YJ (2013). Phase II trial of everolimus for the treatment of nonclear-cell renal cell carcinoma.. Ann Oncol.

[41] Tannir NM, Plimack E, Ng C, Tamboli P, Bekele NB, Xiao L (2012). A phase 2 trial of sunitinib in patients with advanced non-clear cell renal cell carcinoma.. Eur Urol.

[42] Choueiri TK, Vaishampayan U, Rosenberg JE, Logan TF, Harzstark AL, Bukowski RM (2013). Phase II and biomarker study of the dual MET/VEGFR2 inhibitor foretinib in patients with papillary renal cell carcinoma.. J Clin Oncol.

[43] Gore ME, Szczylik C, Porta C, Bracarda S, Bjarnason GA, Oudard S (2009). Safety and efficacy of sunitinib for metastatic renal-cell carcinoma: an expanded-access trial.. Lancet Oncol.

[44] Flanigan RC, Mickisch G, Sylvester R, Tangen C, Van Poppel H, Crawford ED. (2004). Cytoreductive nephrectomy in patients with metastatic renal cancer: a combined analysis.. J Urol.

[45] Hutson TE, Escudier B, Esteban E, Bjarnason GA, Lim HY, Pittman KB (2014). J Clin Oncol.

